# Pre-Pregnancy Risk Factors for Severe Hyperemesis Gravidarum: Korean Population Based Cohort Study

**DOI:** 10.3390/life11010012

**Published:** 2020-12-26

**Authors:** Ho Yeon Kim, Geum Joon Cho, So Yeon Kim, Kyu-Min Lee, Ki Hoon Ahn, Sung Won Han, Soon-Cheol Hong, Hyun Mee Ryu, Min-Jeong Oh, Hai-Joong Kim, Seung Chul Kim

**Affiliations:** 1 Department of Obstetrics and Gynecology, Korea University College of Medicine, 27 Inchonro, Seongbuk-gu, Seoul 02841, Korea; shinbi73@korea.ac.kr (H.Y.K.); hurra7@naver.com (S.Y.K.); akh1220@hanmail.net (K.H.A.); novak082@naver.com (S.-C.H.); mjohmd@korea.ac.kr (M.-J.O.); haijkim@gmail.com (H.-J.K.); 2 School of Industrial Management Engineering, Korea University, 145 Anam-ro, Anam-dong, Seongbuk-gu, Seoul 02841, Korea; clash0830@naver.com (K.-M.L.); swhan@korea.ac.kr (S.W.H.); 3 Department of Obstetrics and Gynecology, CHA Bungdang Medical Center, CHA University School of Medicine, 59 Yatap-ro, Bundang-gu, Seongnam-si, Gyeonggi-do 13496, Korea; hmryu2012@naver.com; 4 Department of Obstetrics and Gynecology, Pusan National University School of Medicine, 2 Busandaehak-ro 63beon-gil, Jangjeon 2(i)-dong, Geumjeong-gu, Busan 46241, Korea

**Keywords:** hospital admission, hyperemesis gravidarum, underweight, alcohol, risk factor

## Abstract

Hyperemesis gravidarum is known to be associated with poor perinatal outcomes. This study aimed to identify pre-pregnancy risk factors for hospital admission in women with hyperemesis gravidarum. We enrolled women who had delivered between 1 January 2013 and 31 December 2015, and had undergone a national health screening examination through the National Health Insurance Corporation 1–2 years before their first delivery. Multiple logistic regression analysis was performed to estimate the risk factors for hospital admission due to hyperemesis gravidarum. Of the 216,373 study participants with hyperemesis gravidarum, 2210 (1.02%) pregnant women were hospitalized. These women had lower waist circumference and were underweight based on body mass index compared to pregnant women who did not require hospitalization due to hyperemesis gravidarum. On multivariate analysis, primiparity, multiple pregnancies, female fetus, alcohol consumption, and pre-pregnancy underweight status were associated with an increased risk of hospitalization due to the condition. In this population-based cohort study, we found that hospitalization due to hyperemesis gravidarum was associated with pre-pregnancy lifestyle characteristics. Early recognition and management of these pre-pregnancy factors may help control the need for hospitalization in women with the condition in subsequent pregnancies.

## 1. Introduction

Hyperemesis gravidarum (HG) is defined as unrelenting nausea and excessive vomiting initiated before the 22nd week of gestation with or without metabolic disturbances, such as electrolyte imbalances, acid-base imbalances, nutritional deficiencies, ketonuria, and weight loss. The incidence of hospitalization for HG is 1–3% among all pregnancies and is the most common cause of hospital admission in the first trimester. HG is known to cause severe perinatal morbidities such as Wernicke’s encephalopathy [1], acute renal failure [2], and fetal growth restriction, preterm birth, and neurodevelopmental delay in the child [3,4]. The symptoms usually cease at the end of the first or early second trimester; however, could extend throughout pregnancy in approximately one-third of the cases, leading to severe weight loss, malnutrition, and dehydration [5]. Furthermore, women with a history of prior admission due to HG have a higher probability of subsequent severe HG [6]. Therefore, physiological, and emotional support may be necessary in the management of severe HG.

Several studies have been conducted on the pathogenesis of HG; however, the causes are not yet well understood. One theory suggests that endocrine factors, such as high human chorionic gonadotropin and estrogen levels cause severe symptoms [7]. HG has also been reported to be associated with hyperthyroidism [8] and female fetuses [9]. A pronounced immune response during pregnancy may result in the condition [10]. Lack of leisure-time physical activity before pregnancy [11], adolescent population and deficiency of some trace elements [12] are also reported to correlate with HG. A recent report suggested higher urokinase plasminogen activator receptor signaling pathway in HG which is one of main components of angiogenesis [13]. However whether this phenomenon is a cause or an effect of HG needs further discussion. A significantly higher incidence of Helicobacter pylori infection was demonstrated in patients with HG than in controls [14]. Lower esophageal sphincter pressure, abnormal gastric and intestinal motilities, abnormal metabolic enzymes, and psychological factors are the possible pathogenetic mechanisms of HG [15].

To the best of our knowledge, only few studies have evaluated the pre-pregnancy factors that could determine the need for hospitalization in women with HG. Previous studies have focused on factors of HG in pregnant women; therefore, it is extremely difficult to identify women in the general population who may be at risk of the condition in subsequent pregnancies. Identifying pre-pregnancy factors of HG could help women better plan their pregnancies and prevent worsening of the condition through early treatment. Early diagnosis and prompt treatment would help alleviate the physical and psychological symptoms in women who are at risk for HG. 

In this study, we assessed the pre-pregnancy risk factors for hospitalization due to HG. Data from health examinations of the majority of the Korean population performed by the Korean National Health Corporation can provide generalizable evidence to determine the pre-pregnancy factors associated with hospitalization in women with HG. 

## 2. Materials and Methods 

The majority of the residents in South Korea have been enrolled in the National Health Insurance Corporation (NHIC) since 2000. Data from the Korea National Health Insurance (KNHI) claims database of the Health Insurance Review and Assessment Service were used in this study. Considering that 97% of the population is obliged to enroll in the KNHI program, their claims database contains information on approximately 50 million Koreans. Moreover, almost complete information of different illnesses has been recorded in this centralized database, with the exception of procedures that are not covered by the insurance, such as cosmetic surgeries.

The NHIC conducts a biannual national health screening examination (NHSE), free of charge; therefore, all insurance subscribers and dependents are encouraged to participate. All the information was provided for this study after de-identification. The Institutional Review Board of Korea University Medical Center (AS17087) approved this study.

Using the KNHI claims database, we identified all women who had delivered between 1 January 2013 and 31 December 2015. Of the 1,590,291 women, we included women who underwent the NHSE 1–2 years before their delivery to evaluate pre-pregnancy characteristics. Hospitalization for HG was identified as the principal or secondary diagnosis using the International Classification of Diseases-10th Revision (ICD-10 code, O21). Since we obtained population data from the KNHI claims database, we could not obtain informed consent from the women. 

Pre-pregnancy factors were evaluated using the NHSE data. The NHSE consists of two components: a health interview and health examination. Data for the following covariates were obtained from the health interviews: age, smoking status, and alcohol consumption. Smoking status was divided into three categories: current smoker, past smoker, and never smoker, based on the answers to “Have you ever smoked cigarettes?” and “If yes, are you a current smoker?” Alcohol consumption was divided into three categories according to the frequency: none per week, one-two times per week, and three or more times per week. Health examination included determination of body mass index (BMI, kg/m^2^). Patients were grouped into three different categories based on their BMI: underweight (<18.5 kg/m^2^), normal (18.5–24.9 kg/m^2^), and obese (≥25 kg/m^2^). Waist circumference (WC) was measured as the narrowest circumference between the lower border of the rib cage and iliac crest when holding breath. Hemoglobin (Hb) levels were measured with a Hitachi 747 Autoanalyzer (Hitachi Instruments Inc., Tokyo, Japan) using enzymatic methods. Primiparity, multiple pregnancies, and sex of the neonates were obtained from the birth records registered in the KNHI claims database. 

Data are expressed as mean ± standard deviation (SD) for continuous variables and as percentages for categorical variables. Clinical and biochemical characteristics between the groups were compared using the t-test for differences in continuous variables and the χ2 test for categorical variables. All tests were two-sided and a *p*-value < 0.05 was considered statistically significant. Adjusted odds ratios (ORs) and 95% confidence intervals (CIs) for hospitalization due to HG were estimated using multivariate logistic regression analysis adjusted for age, family history of hypertension (HTN), smoking history, and obesity based on BMI. Statistical analysis was performed using SAS software, version 9.3 (SPSS Inc., Chicago, IL, USA).

## 3. Results

**Figure 1:**
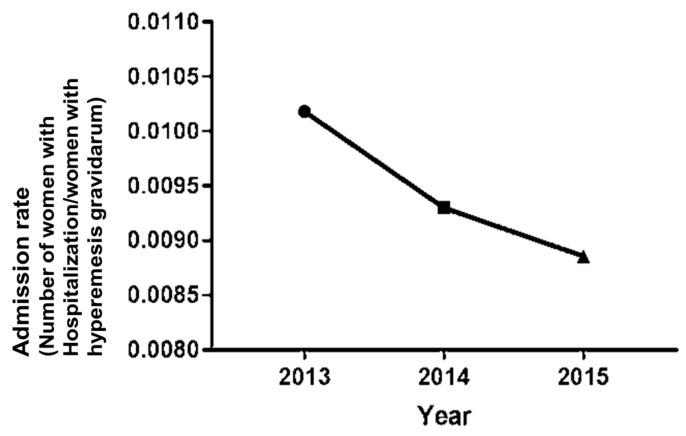
The admission rates of hyperemesis gravidarum.

A total of 216,373 (13.6%) women were diagnosed with HG and had undergone the NHSE 1–2 years before their delivery between 1 January 2013 and 31 December 2015 in the Republic of Korea. In total, 2210 women were hospitalized due to HG (1.02%). Figure 1 demonstrates the annual trend of hospitalization due to HG from 2013 to 2015. The rate of hospitalization decreased significantly (*p* < 0.001). 

A comparison of the demographic and clinical characteristics of the women is presented in Table 1. Women who were hospitalized due to HG were older and had lower pre-pregnancy BMI, WC, and Hb levels. The incidence of hospitalization due to HG was significantly lower in past and current smokers. Women who drank less alcohol before pregnancy experienced more HG than those who consumed it more than once a week. Women with primiparity, multiple pregnancies, and pregnant with a female baby were at a higher risk for hospitalization due to HG.

In the multivariate analysis, primiparity, multiple pregnancies, female sex of the baby, and pre-pregnancy underweight BMI status were statistically associated with an increased risk of hospitalization due to HG (Table 2). Women who consumed alcohol more than once a week before pregnancy were less likely to be hospitalized due to hyperemesis than women who consumed less alcohol (adjusted OR 0.7, 95% CI 0.64–0.76 for alcohol consumption 1–2/week, and adjusted OR 0.64, 95% CI 0.52–0.80 for ≥3/week). However, smoking was not associated with a reduction in the risk. 

## 4. Discussion

This study is one of the largest to evaluate pre-pregnancy factors associated with hospitalization for HG in subsequent pregnancies. Primiparity, multiple pregnancies, female sex of the baby, and pre-pregnancy underweight BMI status were associated with hospital admission due to HG. Women who consumed alcohol more than once a week before pregnancy were less likely to be hospitalized. However, pre-pregnancy smoking habit was not associated with hospitalization due to HG. 

There was no recall bias because the data were recorded during the health examinations, 1 or 2 years before delivery. Ethnic background is a strong confounder in studies on HG. Our results are unlikely to be confounded by ethnicity because the entire population of this study was eastern Asian. The results of the large number of patients with the same ethnicity may indicate generalizability. However, there are some limitations. Physicians in each institution might differ in their decision to hospitalize a patient due to HG. Underweight status with or without poor nutritional intake would encourage physicians to hospitalize the patient. It is also possible that what is regarded as severe vomiting in one population will not be considered severe in another. This outcome measure for hospitalization might be more difficult to assess and subjective than physiological or pathological measurements. The threshold for hospitalization is highly individual; therefore, it may contribute to variability in the diagnosis. Second, the observational design of our study likely limited our ability to identify all the variables, especially socioeconomic status, thyroid function tests, and psychological evaluations, which are not included in the national health examination. Therefore, the reported effects may be overestimated. Lastly, our data included information from 1–2 years before pregnancy; therefore, the actual status at conception was not precisely known. 

The estimated prevalence of hospitalization due to HG of 1.0% in our study was similar and marginally lower than the average prevalence reported in the literature [16,17]. Our study demonstrated a decreased prevalence, possibly due to increased use of drugs for nausea and vomiting [18] and changes in maternal characteristics, such as increasing number of deliveries in older women who especially have lower estrogen levels than younger women [19].

We found that low BMI before pregnancy is a risk factor for hospitalization due to HG. A large retrospective study indicated that women with lean body composition were more likely to be hospitalized due to HG [20,21]. In line with our results, a recent systematic review showed pre-pregnancy BMI, adipose tissue, and leptin correlated with HG in various studies [22]. Less fat deposits might not be capable of neutralizing the circulating placental factors that cause HG. Estrogen is another plausible cause. It has been suggested that women with lower body weight have low circulating estrogen before pregnancy; therefore, might have an exaggerated response to the increase in the hormonal level during the first trimester [7]. Therefore, counseling regarding appropriate weight gain in these women while planning conception would be of great value, and might decrease the incidence of hospitalization due to HG. 

Contrary to a previous research [16], smoking was not associated with HG in our study. Another study using multiple logistic regression analysis after adjusting for age, BMI, and the use of antiemetics demonstrated that smoking significantly aggravated nausea and vomiting in aboriginal Taiwanese women [23]. Moreover, a study conducted on a Chinese population reported that passive smoking increased the risk of vomiting during pregnancy [24]. However, we could not estimate the prevalence of paternal smoking in our study, which could be a confounding factor that may have influenced the results. Another plausible reason is that the prevalence of smoking in our study population was much lower than that in other studies (2.26% vs. 22.8%) [23]. Therefore, studies in a population with a higher prevalence of smoking are needed to explain the association between the habit and HG.

As consistent with previously published reports, primiparity, multiple pregnancies, and female fetus were risk factors for HG [16,17,20,24]. These results are in line with the elevated hCG hypothesis. Significantly higher hCG levels were found in the serum of women bearing female fetuses than in those bearing male fetuses. This also explains the similar prevalence of HG in multiple pregnancies and primigravid women who have markedly elevated hCG levels [25]. These risk factors are not modifiable; therefore, preventive measures are limited.

Among the various pre-pregnancy factors, alcohol consumption was associated with a decreased risk of hospitalization due to HG. However, the reason for this decreased risk is uncertain. To the best of our knowledge, no previous study has investigated this finding. There are several reports that explain the relationship between nausea and vomiting and alcohol consumption. Habitual alcohol consumption demonstrated an inverse correlation with chemotherapy-induced nausea and vomiting, possibly because of genetic polymorphism [26]. Isopropyl alcohol aromatherapy has been used previously to reduce postoperative nausea and vomiting [27]. Beadle et al. demonstrated that inhalation of isopropyl alcohol for 10 min significantly relieved nausea in the emergency department [28]. Another theory is that alcohol mediates estrogen elevation by increased aromatization or decreased catabolism in the liver [29,30]. Estrogen levels are elevated in non-pregnant women who consume alcohol; therefore, the influence of high estrogen during the first trimester might be weaker than that in women who do not consume alcohol. It would be helpful to look for alcohol-related genetic polymorphisms in patients with HG to further elucidate the causal mechanism. Identification of the mechanism of alcohol-related HG might be the key to develop new drugs for the alleviation of hyperemesis. 

Our study implies that appropriate management is necessary for women with HG, including those who do not require hospitalization. Women with severe HG who require hospitalization should be given adequate information and support and should be guided regarding the routine and alternative treatments available to relieve the symptoms. In addition, although we did not assess pregnancy complications following HG with or without hospitalization, previous studies have reported a strong association between severe nausea, vomiting, and preeclampsia/eclampsia in pregnancy. The first sign of abrupt onset of preeclampsia/eclampsia might be severe nausea and vomiting; therefore, differential diagnosis should be considered to prevent adverse outcomes.

## 5. Conclusions

Women can develop dehydration, electrolyte imbalances, weight loss of more than 5% of the pre-pregnancy weight, and psychological trauma such as depression due to pregnancy in cases of severe HG [31]. The goal of our study was to identify the patients at risk of developing severe HG and those requiring hospitalization during pregnancy. To the best of our knowledge, we, for the first time, have demonstrated the pre-pregnancy lifestyle risk factors that predict the need for hospitalization in women with HG. Clinicians should be aware of these factors and consider lifestyle modifications, diet therapy, and alternative treatment modalities for women who are at high risk for HG.

## Figures and Tables

**Table 1:** The pre-pregnancy characteristics of participants stratified by hospitalization for hyperemesis gravidarum.

	No Hospitalization (n = 214,163)	Hospitalization (n = 2210)	*p*-Value
Age (years)	30.51 ± 3.68	30.68 ± 3.77	0.038
Nulliparity (%)	69.96	72.31	0.016
Multiple pregnancies (%)	2.09	5.02	<0.001
Neonatal sex-female (%)	48.41	55.38	<0.001
Smoking history (%)			0.0035
Never	92.85	94.62	
Past	3.76	3.12	
Current	3.39	2.26	
Alcohol (%) (frequency/week)			<0.001
0/week	53.73	63.12	
1–2/week	40.64	32.90	
≥3/week	5.64	3.98	
BMI (kg/m^2^)	±	±	<0.001
Underweight	14.62	17.47	
Normal	75.72	73.89	
Obese	9.66	8.64	
WC (cm)	70.49 ± 7.79	69.87 ± 7.51	<0.001
Hb (mg/dL)	13.02 ± 1.00	12.95 ± 1.01	0.002

Obesity based on BMI: underweight ≤ 18 kg/m^2^, obese ≥ 25 kg/m^2^; values are expressed as mean (SD); BMI, body mass index; HTN, hypertension; WC, waist circumference; Hb, hemoglobin; SD, standard deviation.

**Table 2:** Multiple logistic regression analysis with hospitalization as a dependent variable, and pre-pregnancy factors as independent variables.

	Adjusted ORs	95% CI
Age (years)	0.99	0.98, 1.00
Nulliparity	1.18	1.06, 1.30
Multiple pregnancy	2.43	2.00, 2.95
Neonatal gender-female	1.34	1.23, 1.46
Smoking history (%)		
Never	1	
Past	0.93	0.73, 1.18
Current	0.77	0.58, 1.02
Alcohol (frequency/week)		
0/week	1	
1–2/week	0.70	0.64, 0.76
≥3/week	0.64	0.52, 0.80
BMI (kg/m^2^)		
Underweight	1.16	1.03, 1.31
Normal	1	
Obese	1.04	0.87, 1.24
WC (cm)	1.01	1.00, 1.02
Hb (mg/dL)	1.06	1.00, 1.10

Adjusted for age, family history of HTN, smoking history, and obesity by BMI; OR, odds ratio; CI, confidence interval.

## Data Availability

The KNHI database is not available because it is government-owned database.

## References

[1] Chiossi G., Neri I., Cavazzuti M., Basso G., Facchinetti F. (2006). Hyperemesis gravidarum complicated by Wernicke encephalo-pathy: Background, case report, and review of the literature. Obstet. Gynecol. Surv..

[2] Hill J.B., Yost N.P., Wendel G.D. (2002). Acute Renal Failure in Association with Severe Hyperemesis Gravidarum. Obstet. Gynecol..

[3] Fejzo M.S., Magtira A., Schoenberg F.P., MacGibbon K., Mullin P., Romero R., Tabsh K. (2013). Antihistamines and other prognos-tic factors for adverse outcome in hyperemesis gravidarum. Eur. J. Obstet. Gynecol. Reprod. Biol..

[4] Fejzo M.S., Magtira A., Schoenberg F.P., MacGibbon K., Mullin P.M. (2015). Neurodevelopmental delay in children exposed in utero to hyperemesis gravidarum. Eur. J. Obstet. Gynecol. Reprod. Biol..

[5] Fejzo M.S., Poursharif B., Korst L.M., Munch S., MacGibbon K., Romero R., Goodwin T.M. (2009). Symptoms and Pregnancy Outcomes Associated with Extreme Weight Loss among Women with Hyperemesis Gravidarum. J. Women’s Health.

[6] Trogstad L.I., Stoltenberg C., Magnus P., Skjaerven R., Irgens L.M., Skjærven R. (2005). Recurrence risk in hyperemesis gravidarum. BJOG Int. J. Obstet. Gynaecol..

[7] Rochelson B., Vohra N., Darvishzadeh J., Pagano M. (2003). Low prepregnancy ideal weight:height ratio in women with hy-peremesis gravidarum. J. Reprod. Med..

[8] Hershman J.M. (2004). Physiological and pathological aspects of the effect of human chorionic gonadotropin on the thyroid. Best Pract. Res. Clin. Endocrinol. Metab..

[9] Veenendaal M.V., van Abeelen A.F., Painter R.C., van der Post J.A., Roseboom T.J. (2011). Consequences of hyperemesis gravi-darum for offspring: A systematic review and meta-analysis. BJOG.

[10] Minagawa M., Narita J., Tada T., Maruyama S., Shimizu T., Bannai M., Oya H., Hatakeyama K., Abo T. (1999). Mechanisms un-derlying immunologic states during pregnancy: Possible association of the sympathetic nervous system. Cell Immunol..

[11] Owe K.M., Støer N., Wold B.H., Magnus M.C., Nystad W., Vikanes Å.V. (2019). Leisure-time physical activity before pregnancy and risk of hyperemesis gravidarum: A population-based cohort study. Prev. Med..

[12] Yeh C.-C., Lee F.-K., Wang P.-H. (2018). Hyperemesis gravidarum, pregnancy and bone loss. J. Chin. Med. Assoc..

[13] Yeh C.-C., Tsui K.-H., Wang P.-H. (2018). Hyperemesis gravidarum. J. Chin. Med. Assoc..

[14] Verberg M., Gillott D., Al-Fardan N., Grudzinskas J. (2005). Hyperemesis gravidarum, a literature review. Hum. Reprod. Updat..

[15] Fell D.B., Dodds L., Joseph K.S., Allen V.M., Butler B. (2006). Risk Factors for Hyperemesis Gravidarum Requiring Hospital Admission During Pregnancy. Obstet. Gynecol..

[16] Roseboom T.J., Ravelli A.C., van der Post J.A., Painter R.C. (2011). Maternal characteristics largely explain poor pregnancy out-come after hyperemesis gravidarum. Eur. J. Obstet. Gynecol. Reprod. Biol..

[17] Lee J., Einarson A., Gallo M., Okotore B., Koren G. (2000). Longitudinal change in the treatment of nausea and vomiting of pregnancy in Ontario. Can. J. Clin. Pharmacol. J. Can. Pharmacol. Clin..

[18] Fell D.B., Joseph K.S., Dodds L., Allen A.C., Jangaard K., Van den Hof M. (2005). Changes in maternal characteristics in Nova Sco-tia, Canada from 1988 to 2001. Can. J. Public Health.

[19] Castillo M.J., Phillippi J.C. (2015). Hyperemesis gravidarum: A holistic overview and approach to clinical assessment and man-agement. J. Perinat. Neonatal. Nurs..

[20] Cedergren M., Brynhildsen J., Josefsson A., Sydsjö A., Sydsjö G. (2008). Hyperemesis gravidarum that requires hospitalization and the use of antiemetic drugs in relation to maternal body composition. Am. J. Obstet. Gynecol..

[21] Ioannidou P.G., Papanikolaou D., Mikos T., Mastorakos G., Goulis D.G. (2019). Predictive factors of Hyperemesis Gravidarum: A systematic review. Eur. J. Obstet. Gynecol. Reprod. Biol..

[22] Chou F.-H., Yang Y.-H., Kuo S.-H., Chan T.-F., Yang M.-S. (2009). Relationships among Smoking, Drinking, Betel Quid Chewing and Pregnancy-Related Nausea and Vomiting in Taiwanese Aboriginal Women. Kaohsiung J. Med. Sci..

[23] Zhang J., Cai W.W. (1991). Severe vomiting during pregnancy: Antenatal correlates and fetal outcomes. Epidemiology.

[24] Vikanes Å., Grjibovski A.M., Vangen S., Gunnes N., Samuelsen S.O., Magnus P. (2010). Maternal Body Composition, Smoking, and Hyperemesis Gravidarum. Ann. Epidemiol..

[25] DePue R.H., Bernstein L., Ross R.K., Judd H.L., Henderson B.E. (1987). Hyperemesis gravidarum in relation to estradiol levels, pregnancy outcome, and ther maternal factors: A seroepidemiologic study. Am. J. Obstet. Gynecol..

[26] Uomori T., Horimoto Y., Mogushi K., Matsuoka J., Saito M. (2017). Relationship between alcohol metabolism and chemothera-py-induced emetic events in beast cancer patients. Breast Cancer.

[27] Stoicea N., Gan T.J., Joseph N., Uribe A.A., Pandya J., Dalal R., Bergese S.D. (2015). Alternative Therapies for the Prevention of Postoperative Nausea andVomiting. Front. Med..

[28] Beadle K.L., Helbling A.R., Love S.L., April M.D., Hunter C.J. (2016). Isopropyl Alcohol Nasal Inhalation for Nausea in the Emer-gency Department: A Randoized Controlled Trial. Ann. Emerg. Med..

[29] Couwenbergs C.J. (1988). Acute effects of drinking beer or wine on the steroid hormones of healthy men. J. Steroid Biochem..

[30] Sarkola T., Fukunaga T., Mäkisalo H., Eriksson C.J.P. (2000). Acute effect of alcohol on androgens in premenopausal women. Alcohol Alcohol..

[31] Poursharif B., Korst L.M., Fejzo M.S., MacGibbon K., Romero R., Goodwin T.M. (2007). The psychosocial burden of hyperemesis gravidarum. J. Perinatol..

